# Adult and Pediatric Intra-Institutional Trends of Ciprofloxacin Susceptibility in *E. coli* Positive Urinary Cultures

**DOI:** 10.3390/antibiotics3020163

**Published:** 2014-04-21

**Authors:** Winifred Owumi, Niaz Banaei, Linda D. Shortliffe

**Affiliations:** 1 Department of Urology, Stanford University Medical Center, 750 Welch Rd, MC: 5725, Stanford, CA 94305-5725, USA; E-Mail: isi@stanford.edu; 2 Departments of Pathology and Medicine, Stanford University Medical Center, 300 Pasteur Drive, Stanford, CA 94304, USA; E-Mail: niazbanaei@stanford.edu

**Keywords:** ciprofloxacin, trimethoprim-sulfamethoxazole, nitrofurantoin, susceptibility, bacterial resistance, *E. coli*, urinary cultures, urinary tract infection

## Abstract

Antimicrobial drug resistance in treatment of urinary tract infection (UTI) continues to rise worldwide. To examine contributions of physician prescribing patterns to fluoroquinolone (ciprofloxacin, CP) resistance, we examined *Escherichia coli* (*E. coli*) resistance patterns in urinary cultures. Since CP usage is limited in children, we compared CP resistance trends in adults and children to those of more commonly used trimethoprim-sulfamethoxazole (TMP-SMX) and nitrofurantoin (NF). Our data show that although the general pediatric population has lower resistance to ciprofloxacin, resistance levels are rising with increased usage. While NF susceptibility is historically stable, TMP-SMX resistance is slightly higher in children compared to adults. In both adults and children, antimicrobial resistance patterns vary according to clinical practice site, with ambulatory urology patients showing the highest resistance. This suggests that physician’s prescribing patterns contribute to antimicrobial resistance.

## 1. Introduction

Antimicrobial drug resistance is a major concern in medicine. Urinary tract infections (UTIs) are common in both adults and children and are usually treated empirically. As a result, antimicrobial susceptibility is important for guiding therapy selection. Since susceptibility trends vary among countries, hospitals, clinical practice sites (ambulatory * versus* hospital), and patient groups (adult *versus* child), understanding these trends is important to infection management. While microbial resistance may be innate or acquired from spontaneous or induced mutation, contributions of physician usage patterns on selective pressures are unclear. To better understand this association, we investigated patterns of urinary *Escherichia coli* (*E. coli*) antimicrobial susceptibility within the same institution in different medical specialty practice sites, with the assumption that these sites reflect elements of physician specialists’ usage patterns. Since ciprofloxacin (CP) usage is limited in children, we compared CP resistance in adults and children to two other commonly used antimicrobial agents: trimethoprim-sulfamethoxazole (TMP-SMX) and nitrofurantoin (NF).

## 2. Results

### 2.1. Urinary Cultures

A total of 156,730 urinary cultures were examined. Of these, 38,890 (24.8%) were pediatric cultures (annual average of 3889 ± 230 [SD, Standard deviation]) and 117,840 (75.2%) were adult (annually 11,784 ± 1752). Of these, 7,414 cultures (19.1%) were positive for *E. coli* from the pediatric hospital and 26,582 from adult cultures (22.6%). The majority of these were from females: 5979 (80.2 ± 3.8%) from girls, and 22,950 (86.7 ± 1.6%) from adult females (Table 1). Most urinary specimens positive for *E. coli* were from the ambulatory outpatient clinic and distributed as shown (Table 2).

**Table 1:** Patient demographics (N = number).

Group	Pediatric N	%	Adult N	%
Total positive urine culture	38,890	24.8	117,840	75.2
*E. coli* positive	7414	19.1	26,582	22.6
Females	5979	80.2	22,950	86.7
Males	1416	19.8	3433	13.3
Unknown gender	19	0.2	199	0.8

**Table 2:** *E. coli* positive urinary cultures by clinical site (N = number, OPT = outpatient, INPT = inpatient, ED = emergency department, Gen Peds = general pediatrics, Gen Med = general medicine, OB = obstetrics, GYN = gynecology, URO = urology).

Group	Pediatric N	%	Adult N	%
All Treatment Sites	7414	19.1	26,582	22.6
OPT	6245	84.2	22,868	86
INPT	639	8.6	3709	14
ED	1719	23.2	5444	20.5
Gen Peds/Gen Med	1267	17.1	12,616	47.5
OB/GYN	698	9.4	917	3.4
URO	283	3.8	1703	6.4

### 2.2. Distribution of Antibiotics Susceptibilities

**Figure 1:**
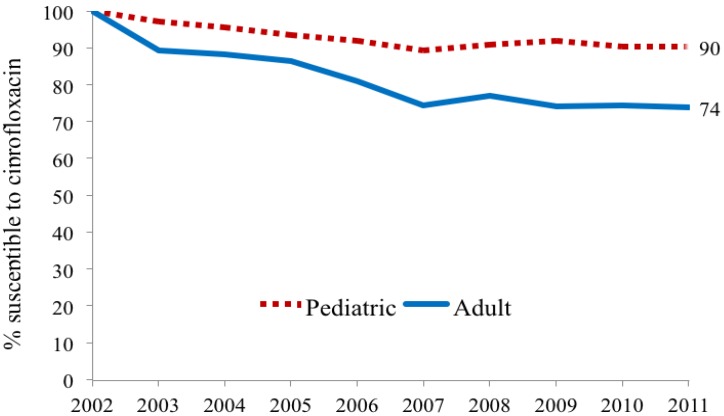
Adult and pediatric ciprofloxacin susceptibility trends for *E. coli* positive urinary cultures.

**Figure 2:**
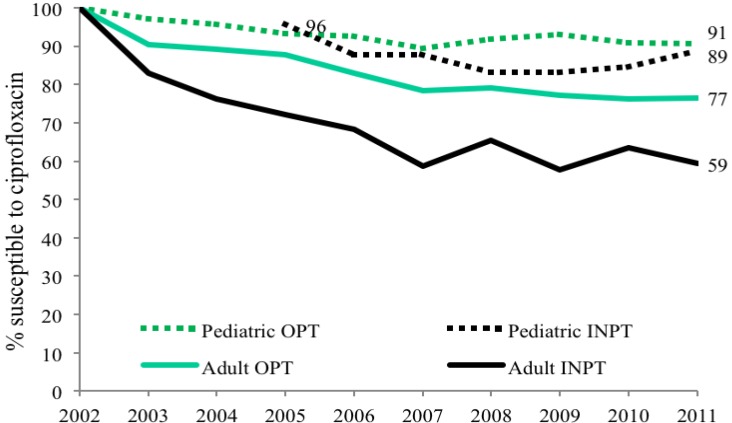
Outpatient and inpatient adult and pediatric ciprofloxacin susceptibility trends for *E. coli* positive urinary cultures (OPT = outpatient, INPT = inpatient).

**Figure 3:**
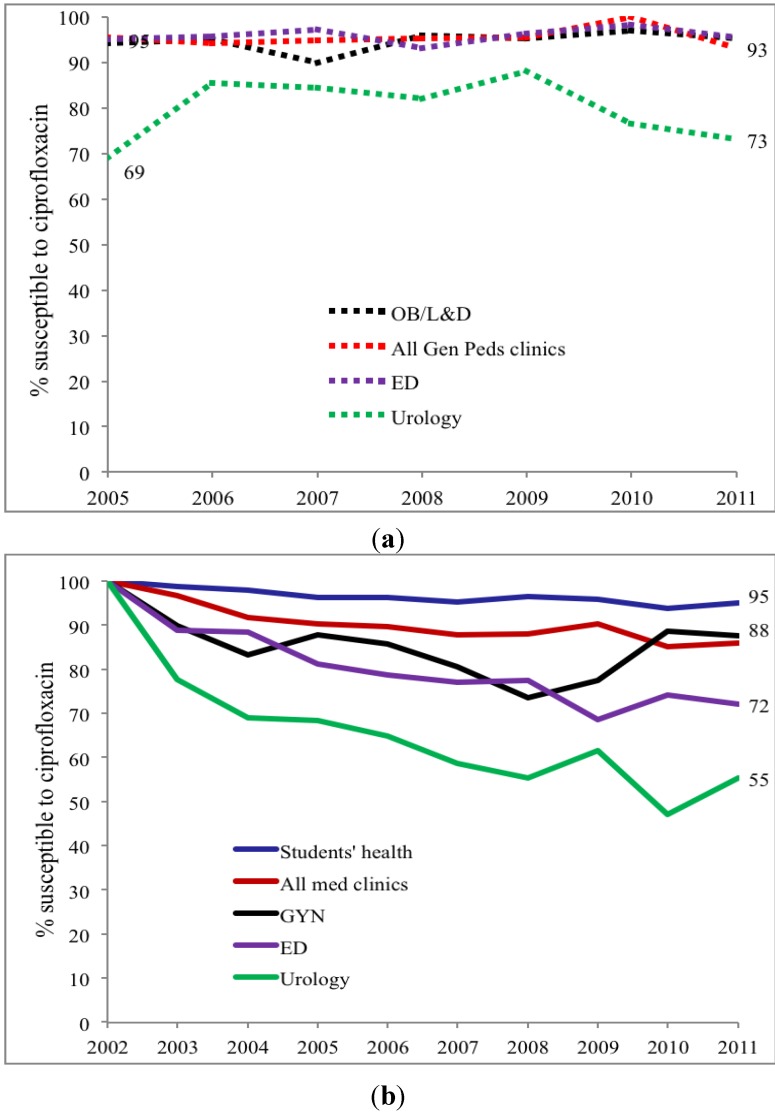
(**a**) *Outpatient pediatric ciprofloxacin* susceptibility trends for *E. coli* positive urinary cultures (ED = emergency department, All Gen Peds clinics = general pediatrics clinics, OB = Obstetrics clinics/Labor and delivery, URO = urology clinics); (**b**) *Outpatient adult ciprofloxacin* susceptibility trends for *E. coli* positive urinary cultures (Students’ health = undergraduate and graduate students’ clinic, ED = emergency department, All med clinics = general medicine clinics, GYN = gynecology clinics, URO = urology clinics).

Urinary *E. coli* was more likely to be susceptible to ciprofloxacin (CP) in children compared to adults (*p* < 0.002). Both child and adult sites showed decreasing susceptibilities from 100% susceptibility in 2002 to 90% in 2011 in children, as compared to 74% in adults (Figure 1). Outpatient (OPT) urine cultures had higher susceptibility to CP than inpatient specimen in both children and adults, with a larger difference in adults (Figure 2). Susceptibility also varied among ambulatory sites, with the lowest susceptibilities in the Urology clinic specimens (73% in children) as compared to other sites that remained between 88 and 95%. Adult urology ambulatory specimens showed a drop in susceptibility to 55% by 2011 (Table 3 and Table 4 and Figure 3a). Similarly by 2011 inpatient hospital specimens were only 60% susceptible to ciprofloxacin (Table 4 and Figure 3b). All *p*-values were <0.02.

**Table 3:** Ciprofloxacin susceptibilities of **pediatric** *E. coli* positive urinary cultures by site (N = number, OPT = outpatient, INPT = inpatient, ED = emergency department, Gen Peds = general pediatrics, OB = obstetrics, URO = urology).

Year	All *E. coli* Cultures N	All *Sites* N (%)	OPT N (%)	INPT N (%)	ED N (%)	Gen Peds N (%)	OB N (%)	URO N (%)
2002	528	490 (100)	N/A	N/A	93 (100)	N/A	N/A	N/A
2003	577	519 (97)	N/A	N/A	109 (100)	N/A	N/A	N/A
2004	1074	861 (95.5)	N/A	N/A	113 (99.1)	N/A	N/A	N/A
2005	1144	968 (93.5)	876 (93.4)	90 (95.7)	114 (95)	499 (95.6)	94 (94)	20 (69)
2006	680	584 (92)	526 (92.4)	58 (87.9)	132 (95.7)	146 (94.2)	94 (94.9)	29 (85.3)
2007	632	520 (89.2)	448 (89.4)	72 (87.8)	141 (97.2)	112 (94.9)	88 (89.8)	32 (84.2)
2008	631	518 (90.9)	464 (91.9)	54 (83.1)	162 (93.1)	103 (95.4)	93 (95.9)	18 (81.8)
2009	749	623 (92)	563 (93.1)	60 (83.3)	255 (96.2)	85 (95.5)	78 (95.1)	29 (87.9)
2010	731	571 (90.2)	510 (90.9)	61 (84.7)	224 (98.2)	74 (100)	93 (96.9)	29 (76.3)
2011	668	554 (90.4)	468 (90.7)	87 (88.8)	189 (95.5)	94 (93.1)	80 (95.2)	27 (73)
Total	7414	6208	5718	489	1532	1482	620	198
Mean	741	621 (93)	572 (93)	61 (87)	153 (97)	185 (96)	89 (95)	20 (80)
SD	205	162 (3)	158 (3)	26 (4)	54 (2)	159 (2)	7 (2)	11 (7)

**Table 4:** Ciprofloxacin susceptibilities of **adult** *E. coli* positive urinary cultures by site (N = number, OPT = outpatient, INPT = inpatient, ED = emergency department, Gen Med = general medicine, GYN = gynecology, URO = urology).

Year	All *E. coli* Cultures N	All *Sites* N (%)	OPT N (%)	INPT N (%)	ED N (%)	Gen Med N (%)	GYN N (%)	URO N (%)
2002	1557	1306 (100)	1083 (100)	223 (100)	268 (100)	470 (100)	70 (100)	123 (100)
2003	1538	1233 (89.2)	1061 (90.3)	168 (82.8)	333 (88.8)	501 (96.7)	44 (89.8)	101 (77.7)
2004	3484	2683 (88.2)	2500 (89.2)	183 (76.3)	344 (88.4)	1953 (91.8)	30 (83.3)	104 (68.9)
2005	4456	3518 (86.4)	3270 (87.7)	248 (72.1)	338 (81.3)	2700 (90.4)	50 (87.7)	97 (68.3)
2006	2425	1817 (81)	1599 (83.1)	218 (68.3)	376 (78.7)	949 (89.6)	48 (85.7)	96 (64.9)
2007	2298	1589 (74.5)	1345 (78.3)	244 (58.7)	367 (77.1)	722 (87.8)	62 (80.5)	98 (58.7)
2008	2540	1816 (76.9)	1571 (79.1)	245 (65.5)	454 (77.5)	825 (88)	67 (73.6)	89 (55.3)
2009	2702	1852 (74.1)	1631 (77.1)	221 (57.7)	470 (68.5)	841 (90.3)	79 (77.5)	96 (61.5)
2010	2425	1847 (74.3)	1621 (76.1)	226 (63.5)	466 (74.1)	789 (85.2)	116 (88.5)	71 (47)
2011	2857	1955 (74)	1712 (76.6)	239 (59.5)	499 (72.1)	770 (85.9)	141 (87.6	92 (55.4)
Total	26582	19616	17396	2216	3715	10520	707	967
Mean	2658	1962 (82)	1740 (84)	222 (70)	392 (81)	1052 (91)	71 (85)	97 (66)
SD	858	676 (9)	670 (8)	27 (13)	76 (9)	710 (5)	34 (7)	13 (15)

**Figure 4:**
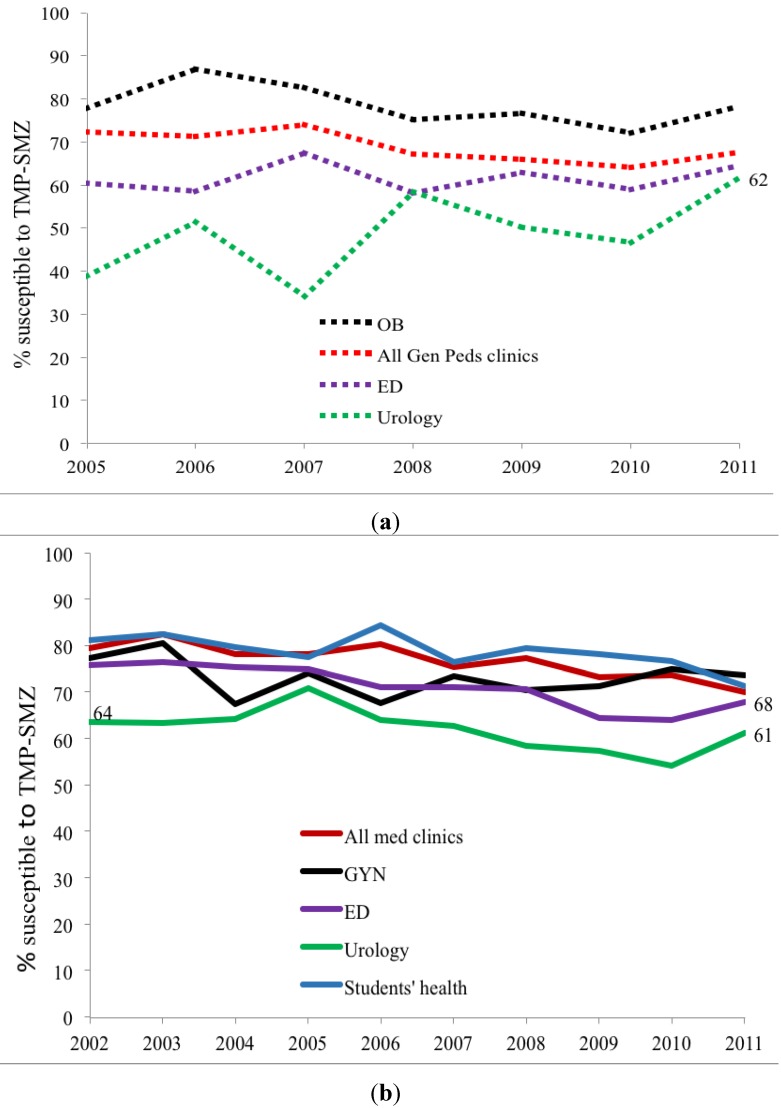
(**a**) *Outpatient pediatric TMP-SMX* susceptibility trends for *E. coli* positive urinary cultures (ED = emergency department, All Gen Peds clinics = general pediatrics clinics, OB=obstetrics clinics/Labor and delivery, URO=urology clinics); (**b**) *Outpatient adult TMP-SMX* susceptibility trends for *E. coli* positive urinary cultures (Students’ health = undergraduate and graduate students’ clinic, ED = emergency department, All med clinics = general medicine clinics, GYN = gynecology clinics, URO = urology clinics).

**Figure 5:**
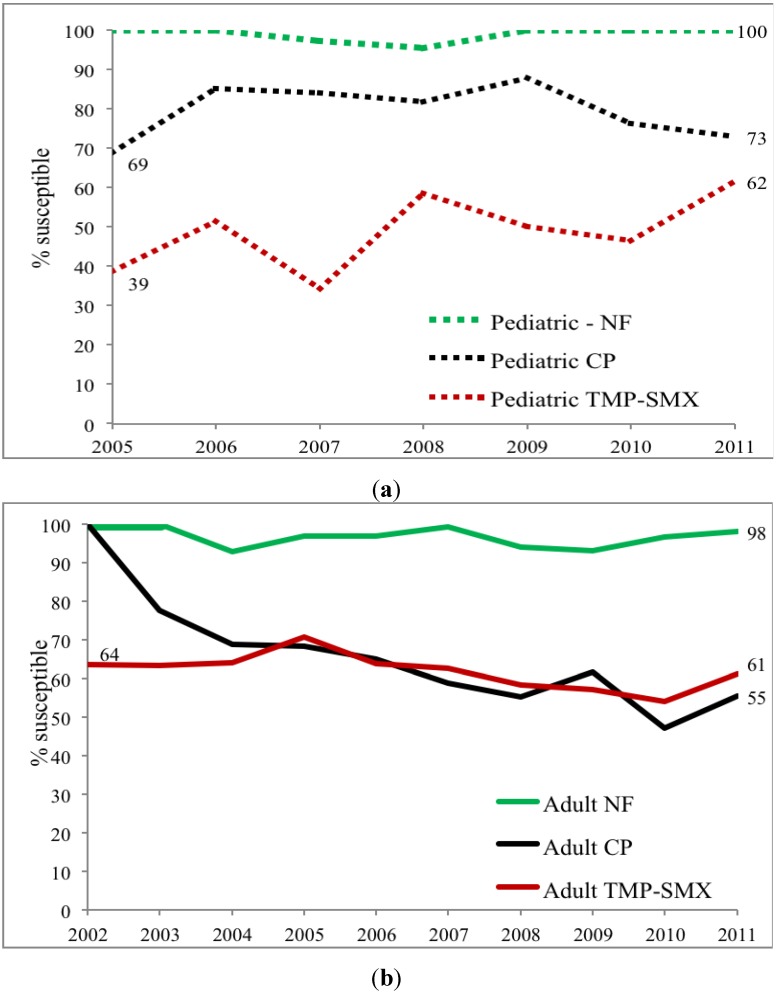
(**a**) *Pediatric urology clinic* susceptibility trends for three commonly prescribed antimicrobial agents for *E. coli* positive urinary cultures (NF = nitrofurantoin, CP = ciprofloxacin, TMP-SMX = trimethoprim-sulfamethoxazole); (**b**) *Adult urology clinic* susceptibility trends for three commonly prescribed antimicrobial agents for *E. coli* positive urinary cultures (NF = nitrofurantoin, CP = ciprofloxacin, TMP-SMX = trimethoprim-sulfamethoxazole).

Trimethoprim-sulfamethoxazole (TMP-SMX) susceptibility is lower in children than adults. While TMP-SMX susceptibility declined during the study period, the rate of decrease was lower than for CP. Again, susceptibility varied with clinical treatment site and lowest susceptibility occurred in pediatric and adult urology ambulatory sites (Figure 4a,b, *p* < 0.02). *E. coli* NF susceptibility for both children and adult specimens was, meanwhile, relatively stable varying from 93%–100% between 2002 and 2011 without significant difference between adults and children nor among treatment sites. Comparison of these three commonly used antibiotics within ambulatory urology demonstrated three patterns of *E. coli* susceptibility: (a) no differences in NF susceptibility between children and adults; (b) lower TMP-SMX susceptibilities in children than adults; and (c) higher CP susceptibility in children than adults Figure 2, Figure 3, Figure 4 and Figure 5).

## 3. Discussion

Simultaneous to development of powerful and less toxic antimicrobial agents, infecting organisms are developing more resistance to antimicrobial agents. This has been a trend that has been reported over the last 20 years, as widespread usage of antimicrobial agents is believed to create selection pressure for more resistant organisms [1]. Case-control studies comparing children with and without TMP-SMX resistant organisms have shown that children who had antimicrobials agents for greater than four weeks in the previous six months were 23 times more likely to have resistance compared with those without resistance; children with genitourinary abnormalities were 2.4 times more likely to have resistance; and children with previous hospital admissions 2.3 times more likely [2] In other cases, specific clones of multidrug-resistant *E. coli* have been shown to behave with “epidemic outbreak” characteristics. The sources of these clonal epidemic-like outbreaks are unclear; some evidence implicates food and/or contact with food sources that had antimicrobial agent contact [3]. While international, national, regional, local, or hospital-wide antibiograms reflecting organism resistance attract attention [4,5], there is limited research on organism resistance and susceptibility diversity by practice site within institutions.

In this study we document that: (1) *E. coli* antimicrobial susceptibility differs widely between adults and children; (2) further variation occurs by practice site within a single medical center; and (3) differing drug trends in susceptibilities may occur. We hypothesize that these practice site susceptibility differences reflect the differing antimicrobial practice patterns of specialists and their specialty practice patterns. These antimicrobial drug trends may show: (a) differing susceptibilities between adults and children; (b) similar decreasing antimicrobial drug susceptibilities for both adults and children; and (c) persistently low susceptibilities for both. Our findings suggest that antimicrobial susceptibility and resistance patterns are affected by specialty practice patterns.

This study shows that pediatric and adult *E. coli* UTI susceptibility trends differ for three commonly used antimicrobial agents. With limited usage in children overall CP susceptibilities (69%–88%) are high in children except for the lower levels in ambulatory urology with higher usage; in contrast results show lower susceptibility in adults with lowest levels in adult ambulatory urology. A decreasing susceptibility rate for *E. coli* in urology practices has been noted by others in Japan [6]. The widespread usage of TMP-SMX in children for otitis media in addition to UTI is reflected in overall *E. coli* susceptibilities of 39%–62% while adult susceptibilities range from 61%–71%. The stable higher *E. coli* susceptibilities of NF for both adults (>92%) and children (>95%) for the study periods most likely reflect relatively limited usage of NF for lower urinary tract infections and prophylaxis as reported by others [7]. By combining data from multiple institutional and internal practice sites, others have not revealed the range of *E. coli* susceptibilities within a single site nor the differences between children and adults [8]. Our data show that using individual hospital or institutional antibiograms to guide empiric antimicrobial treatment of a UTI in child or adult, moreover, may be misleading (Figure 1 and Figure 2 and Table 3 and Table 4).

These data and conclusions are limited by several considerations. First, prior to 2005 the sources of our pediatric specimens were not separated by outpatient and inpatient locations, so data trends are from 2005 forward, and second, CP patterns were not recorded in 2002 for technical reasons. Because data were de-identified, we could not eliminate repeat specimen data for patients. While multiple specimens from individuals could decrease or increase our susceptibility calculations, we believe that the large number of specimens involved reflect valid trends. Our usage of threshold of 10,000 CFU/ml as a positive culture may have excluded some urinary specimens with fewer resistant organisms, and this could overestimate our susceptibility reports. Finally, these studies compare data from an institution that offers primary medical care to the surrounding community in addition to regional and national tertiary care, thus these data may be unique based upon this patient population.

## 4. Experimental

After Stanford University Medical Center Institutional Review Board approval was obtained for this study, we performed a retrospective review of all urine cultures submitted to Stanford University Medical Center (SUMC) Microbiology Laboratory from 2002 to 2011. Cultures were from Stanford University Hospitals and Clinics (SUH) reflecting specimens obtained primarily from adults, and from Lucile Packard Children’s Hospital and Clinics (LPCH) from children and adult women seen in obstetrics clinic (OB) or admitted to labor and delivery (L&D). Data included inpatient (INPT) and outpatient (OPT) urinary specimens obtained by all collection methods; repeat specimens per individual could not be excluded because data were de-identified. Microbiological data were obtained from two sources: (1) the SUMC clinical microbiology laboratory electronic database that contains urinary culture and susceptibility reports and annual hospital-wide antimicrobial susceptibility data; and (2) the STRIDE database (*Stanford Translational Research Integrated Database Environment* that compiles annual urinary culture specimen numbers, including positive and negative results. This is an NIH (National Institutes of Health) sponsored program that compiles clinical data for research use from SUMC. From the annual microbiology laboratory reports, hospital-wide trends were compiled for both the adult and pediatric hospitals (“antibiograms”). These antibiogram reports include cultures and susceptibilities from any type of culture (example, urine, blood, tracheal aspirates, *etc.*) processed at the SUMC laboratory.

STRIDE total urine cultures data were screened for selection using UTI diagnosis based on the following ICD-9 codes: 599.0 (UTI site not specified), 595 (cystitis), 590.1 (Acute pyelonephritis), 590.8 (Pyelonephritis unspecified). These data were segregated between the adult and pediatric hospitals by using an age cutoff of 18 years and below for pediatric patient data. This method generated total numbers of urinary cultures sent for populations. Antibiotic urinary susceptibility data was retrieved from the microbiology laboratory database that stores data for adult and pediatric hospitals separately. All SUMC clinical microbiology laboratory electronic urinary culture data were first parsed for overall antimicrobial susceptibility trends, then separated between INPT and OPT. The OPT clinical site data were further subdivided to various ambulatory clinics. For the adult hospital the OPT sites included the ED (emergency department), Gen Med (all internal medicine clinics including Stanford family medicine, Stanford medical group, Stanford family medicine, off-site community practice medical clinics using the Stanford laboratory), GYN (Gynecology) and URO (Urology). The pediatric OPT sites included the ED, Gen Peds (General pediatric clinics, including onsite and offsite community practice groups), OB (Obstetrics/Labor and delivery) and URO (Urology). Of note, except for the ED, prior to 2005, the pediatric hospital data entry did not code separately for outpatient specialty clinic sites.

Urinary specimens with *E. coli* were considered positive at a threshold of 10,000 cfu/mL (colony forming units per milliliter) in purity or predominance. This threshold was chosen because antimicrobial susceptibilities are routinely reported for 10,000 CFU/mL and we were examining *E. coli* susceptibilities in urinary cultures not urinary tract infection rates. Antimicrobial susceptibilities were performed using a Vitek2 microbial identification system (bioMérerieux Inc., Durham, North Carolina, USA) and MICs were interpreted according to the Clinical and Laboratory Standard Institute M100 document.

### Statistical Analysis

We calculated the percentage annual urinary culture susceptibility as the numbers of specimens sensitive to the specific antibiotic divided by the annual *E. coli* positive cultures (CP, TMP-SMX and NF). The figures obtained were plotted annually to obtain the annual trend corresponding to the various clinical sites evaluated. Independent (unpaired) two-tailed Student’s *t*-test (*p*-value) were calculated for comparison of adult *versus* pediatric sites susceptibility to assess the statistical significance of differences seen among clinical sites in adults and children.

## 5. Conclusions

We report that antimicrobial susceptibilities for *E. coli* urinary cultures from a single institution may differ significantly between adults and children for three commonly used agents. Differences may occur, furthermore, among various practice sites within a single hospital site. We suggest that these differences in susceptibility reflect distinct specialty clinical prescribing practices. As a result of these variations, reliance upon any single regional or local antibiograms within large practice medical centers could be misleading.
